# Supplementary material for: Formation of memory assemblies through the DNA-sensing TLR9 pathway
Source: Nature. 2024 Mar 27;628(8006):145–53. doi: 10.1038/s41586-024-07220-7 (PMC10990941; doi:10.1038/s41586-024-07220-7)
Supplement: Supplementary file 1 — Gating strategy for FACS of GFP+ nuclei. [file 41586_2024_7220_MOESM1_ESM.pdf]

nature portfolio
https://doi.org/10.1038/s41586-024-07220-7

Supplementary information

Formation of memory assemblies through the DNA-sensing TLR9 pathway

In the format provided by the authors and unedited

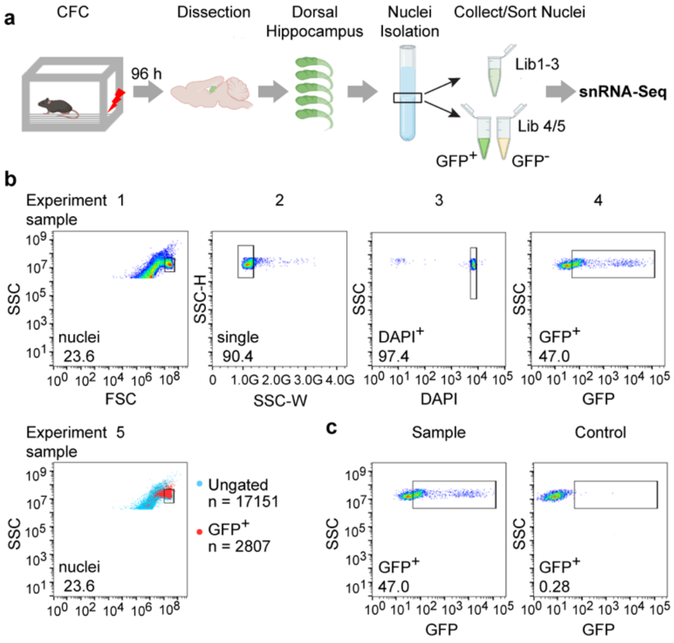

Supplemental Data Fig. 1: Nuclei Gating Strategy. a Sample preparation **s**chematic. **b** Example of an experimental sample. 1) Gated on the nuclei population based on the FSC/SSC distribution. 2) Plot SSC-W/SSC-H to remove the aggregates. 3) Gated on DAPI+ nuclei, further getting grid of aggregates. 4) Define the GFP+ population. 5) Overlay GFP+ with ungated population to verify the accuracy of nuclei gating **c** GFP+ gate is based on the control of GFP- sample.
