# Supplementary material for: Formation of memory assemblies through the DNA-sensing TLR9 pathway
Source: Nature. 2024 Mar 27;628(8006):145–53. doi: 10.1038/s41586-024-07220-7 (PMC10990941; doi:10.1038/s41586-024-07220-7)
Supplement: Supplementary file 2 — Reporting Summary [file 41586_2024_7220_MOESM2_ESM.pdf]

nature portfolio

Corresponding author(s): Jelena Radulovic

Last updated by author(s): February 4, 2004

Reporting Summary

Nature Portfolio wishes to improve the reproducibility of the work that we publish. This form provides structure for consistency and transparency in reporting. For further information on Nature Portfolio policies, see our Editorial Policies and the Editorial Policy Checklist.

Statistics

For all statistical analyses, confirm that the following items are present in the figure legend, table legend, main text, or Methods section.

n/a	Confirmed	
☐	☒	The exact sample size (*n*) for each experimental group/condition, given as a discrete number and unit of measurement
☐	☒	A statement on whether measurements were taken from distinct samples or whether the same sample was measured repeatedly
☐	☒	The statistical test(s) used AND whether they are one- or two-sided<br>*Only common tests should be described solely by name; describe more complex techniques in the Methods section.*
☐	☒	A description of all covariates tested
☒	☐	A description of any assumptions or corrections, such as tests of normality and adjustment for multiple comparisons
☐	☒	A full description of the statistical parameters including central tendency (e.g. means) or other basic estimates (e.g. regression coefficient) AND variation (e.g. standard deviation) or associated estimates of uncertainty (e.g. confidence intervals)
☐	☒	For null hypothesis testing, the test statistic (e.g. *F*, *t*, *r*) with confidence intervals, effect sizes, degrees of freedom and *P* value noted<br>*Give P values as exact values whenever suitable.*
☒	☐	For Bayesian analysis, information on the choice of priors and Markov chain Monte Carlo settings
☒	☐	For hierarchical and complex designs, identification of the appropriate level for tests and full reporting of outcomes
☐	☒	Estimates of effect sizes (e.g. Cohen's *d*, Pearson's *r*), indicating how they were calculated

Our web collection on statistics for biologists contains articles on many of the points above.

Software and code

Policy information about availability of computer code

Data collection	snRNA-seq libraries were generated from about 8,000 individual cells captured in an oil emulsion on a Chromium Controller (10x Genomics, Pleasanton, CA.) cDNA was generated in the individual cell-gel bead emulsion micro-reactors while adding barcodes at the cellular and molecular level using the Chromium Next GEM Single Cell 3' Kit v3.1(10x Genomics kit 1000268.) The barcoded cDNAs from the individual cells were combined for the remaining library process. The unique molecular barcodes (UMIs) ensure that amplification artifacts will not skew the analysis. The libraries were analyzed on a Fragment Analyzer 5200 (Agilent Technologies, Santa Clara, CA) to ensure a normal size distribution with an average size of 450 bp. The libraries were sequenced on an llumina Sequencer with the following read lengths: 28 bp for Read 1, 10bp for i7 Index, 10bp for i5 Index and 90 bp for Read 2 at a read depth of 20,000 reads per cell.
Data analysis	Statistical analysis:<br>GraphPad Prism Version 10.1.0<br>Power analysis calculator: http://www.stat.ubc.ca/~rollin/stats/ssize/n2.html<br><br>Sequencing Data Analysis:<br>SAMtools flagstat<br><br>Gene Expression Analysis:<br>STRING database (version 11.0)<br>GeneMANIA prediction serve<br>Mouse Genome Database (MGI) Batch Query Genome Analysis Tool (v6.23)<br>PANTHER Classification System engine, version 17.0<br>reactome.org, Version 86

R:
MEDIPS R package
Cellranger software v7.0.1
Seurat R package v4.3.0
scDblFinder v1.13.13
SoupX v1.6.2
Seurat SCTransform function
fgsea v1.20.0
Images:
Fiji Image J (NIH, vl.53f51, RRID:SCR_003070)

Figures:
Adobe Illustrator CS6 (Adobe, v27.5, RRID: SCR_010279)
Biorender.com

Code: GitHub (https://github.com/RadulovicLab/Nature-2024)

For manuscripts utilizing custom algorithms or software that are central to the research but not yet described in published literature, software must be made available to editors and reviewers. We strongly encourage code deposition in a community repository (e.g. GitHub). See the Nature Portfolio guidelines for submitting code & software for further information.

Data

Policy information about availability of data

All manuscripts must include a data availability statement. This statement should provide the following information, where applicable:

- Accession codes, unique identifiers, or web links for publicly available datasets
- A description of any restrictions on data availability
- For clinical datasets or third party data, please ensure that the statement adheres to our policy

The bulk RNA-seq data are available at the NCBI Gene Expression Omnibus database under accession numbers GEO: GSE174076. The snRNA-seq data are available at the NCBI Gene Expression Omnibus database under accession numbers GEO: GSE254780. Source Data are provided with this paper. The mouse genome mus musculus mm10 was referenced for snRNA-seq. shRNA sequences were provided at GitHub (https://github.com/RadulovicLab/Nature-2024)

Human research participants

Policy information about studies involving human research participants and Sex and Gender in Research.

Reporting on sex and gender	N/A
Population characteristics	N/A
Recruitment	N/A
Ethics oversight	N/A

Note that full information on the approval of the study protocol must also be provided in the manuscript.

Field-specific reporting

Please select the one below that is the best fit for your research. If you are not sure, read the appropriate sections before making your selection.

☒ Life sciences ☐ Behavioural & social sciences ☐ Ecological, evolutionary & environmental sciences

For a reference copy of the document with all sections, see nature.com/documents/nr-reporting-summary-flat.pdf

Life sciences study design

All studies must disclose on these points even when the disclosure is negative.

Sample size	Statistical power to detect anticipated effect sizes was determined using power analysis (calculator at http://www.stat.ubc.ca/~rollin/stats/ssize/n2.html) conducted on representative samples of previous work and pilot experiments. For all proposed experiments, minimum power is set at 0.90 to detect an alpha = 0.05 (2 - sided test) for a difference in means from 20% to 40%, with a 15% common standard deviation.

Data exclusions	Mice with misplaced virus infusion or cannulas, and mice with less than 70% viral expression in the CAl were excluded. These exclusions are pre-established within the field and previously published by our lab and others. Cells with less than 1000 detected genes, more than 4000 detected genes, or larger than 5 percent mitochondrial gene percentage were excluded for further analysis, as according to established exclusion criteria for snRNA-seq.
Replication	All significant behavioral effects were replicated at least 3 times using WT, virus specific, and cell specific control groups. All cellular and molecular effects were shown in 6-12 biological replicates. All main gene expression effects were replicated with four different approaches (bulk RNA-seq, qPCR arrays, qPCR, and snRNA-seq). Replicates produced similar results relative to initial/representative experiments.
Randomization	With all genetic lines bred in our facility, littermates were randomly assigned to different experimental groups to minimize litter effects.
Blinding	The behavioral tests were performed blindly, either by experimentalists who were unaware of the treatments because the solutions were coded or by experimenters unaware of the experimental design. The experimenter performing the tests was not aware of the numbering code. Immunohistochemical analyses were performed by automated software (ImageJ).

Reporting for specific materials, systems and methods

We require information from authors about some types of materials, experimental systems and methods used in many studies. Here, indicate whether each material, system or method listed is relevant to your study. If you are not sure if a list item applies to your research, read the appropriate section before selecting a response.

Materials & experimental systems

n/a	Involved in the study
☐	☒ Antibodies
☐	☒ Eukaryotic cell lines
☒	☐ Palaeontology and archaeology
☐	☒ Animals and other organisms
☒	☐ Clinical data
☒	☐ Dual use research of concern

Methods

n/a	Involved in the study
☒	☐ ChIP-seq
☐	☒ Flow cytometry
☒	☐ MRI-based neuroimaging

Antibodies

Antibodies used	Please see **Supplementary Methods** summarizing all relevant details and specifying recombinant, knock-out validated, or enhanced validation. For all other antibodies, we used pre-absorbtion or secondary only antibody controls. For the DNase antibody, we used DNase treatment as a control, for Tlr9 and RelA we used knockout tissue as controls.
Validation	Please see **Supplementary Methods** for a full list of primary antibodies with validation methods and/or relevant citations.

Eukaryotic cell lines

Policy information about cell lines and Sex and Gender in Research

Cell line source(s)	*State the source of each cell line used and the sex of all primary cell lines and cells derived from human participants or vertebrate models.*
Authentication	*Describe the authentication procedures for each cell line used OR declare that none of the cell lines used were authenticated.*
Mycoplasma contamination	*Confirm that all cell lines tested negative for mycoplasma contamination OR describe the results of the testing for mycoplasma contamination OR declare that the cell lines were not tested for mycoplasma contamination.*
Commonly misidentified lines (See ICLAC register)	*Name any commonly misidentified cell lines used in the study and provide a rationale for their use.*

Palaeontology and Archaeology

Specimen provenance	*Provide provenance information for specimens and describe permits that were obtained for the work (including the name of the issuing authority, the date of issue, and any identifying information). Permits should encompass collection and, where applicable, export.*
Specimen deposition	*Indicate where the specimens have been deposited to permit free access by other researchers.*

Dating methods | *If new dates are provided, describe how they were obtained (e.g. collection, storage, sample pretreatment and measurement), where they were obtained (i.e. lab name), the calibration program and the protocol for quality assurance OR state that no new dates are provided.*

☐ Tick this box to confirm that the raw and calibrated dates are available in the paper or in Supplementary Information.

Ethics oversight | *Identify the organization(s) that approved or provided guidance on the study protocol, OR state that no ethical approval or guidance was required and explain why not.*

Note that full information on the approval of the study protocol must also be provided in the manuscript.

Animals and other research organisms

Policy information about studies involving animals; ARRIVE guidelines recommended for reporting animal research, and Sex and Gender in Research

Laboratory animals	Eight-week-old male and female C57BL/6N mice were obtained from Envigo. Tlr9$^{flox/flox}$ (C57BL/6J-Tlr9em1Ldm/J) mice with *loxP* sites flanking exon 1 of theTlr9, IFNAR$^{flox/flox}$ (B6(Cg)-Ifnar1tm1.1Ees/J) mice with *loxP* sites flanking exon 3 of the type-1 interferon α/β receptor gene, RelA$^{flox/flox}$ (B6.129S1-Relatm1Ukl/J) mice with *loxP* sites flanking exon 1 of the Rela gene, and STING knockout mice C57BL/6J-Sting1gt/J; Goldenticket (Tmem173gt) were obtained from Jackson Laboratory and bred in institutional facilities. All mice were 8 weeks of age at the beginning of experiments. All mice were group housed (12 h light/dark cycle with lights on at 7 AM, temperature 20-22°C, humidity 30-60%) with ad libitum access to food and water (mice were switched to single housing one-week prior to experimentation).
Wild animals	No wild animals were used in this study.
Reporting on sex	All experimental groups were mixed sex, consisting of ~equal number of males and females.
Field-collected samples	No field collected samples were used in this study.
Ethics oversight	All procedures were approved by Northwestern University's Animal Care and Use Committee and Albert Einstein's Animal Care and Use Committee in compliance with US National Institutes of Health standards, and Aarhus University in compliance with the Danish National Animal Experiment Committee.

Note that full information on the approval of the study protocol must also be provided in the manuscript.

Clinical data

Policy information about clinical studies

All manuscripts should comply with the ICMJE guidelines for publication of clinical research and a completed CONSORT checklist must be included with all submissions.

Clinical trial registration	*Provide the trial registration number from ClinicalTrials.gov or an equivalent agency.*
Study protocol	*Note where the full trial protocol can be accessed OR if not available, explain why.*
Data collection	*Describe the settings and locales of data collection, noting the time periods of recruitment and data collection.*
Outcomes	*Describe how you pre-defined primary and secondary outcome measures and how you assessed these measures.*

Dual use research of concern

Policy information about dual use research of concern

Hazards

Could the accidental, deliberate or reckless misuse of agents or technologies generated in the work, or the application of information presented in the manuscript, pose a threat to:

No	Yes	
☒	☐	Public health
☒	☐	National security
☒	☐	Crops and/or livestock
☒	☐	Ecosystems
☒	☐	Any other significant area

Experiments of concern

Does the work involve any of these experiments of concern:

No	Yes	
☒	☐	Demonstrate how to render a vaccine ineffective
☒	☐	Confer resistance to therapeutically useful antibiotics or antiviral agents
☒	☐	Enhance the virulence of a pathogen or render a nonpathogen virulent
☒	☐	Increase transmissibility of a pathogen
☒	☐	Alter the host range of a pathogen
☒	☐	Enable evasion of diagnostic/detection modalities
☒	☐	Enable the weaponization of a biological agent or toxin
☒	☐	Any other potentially harmful combination of experiments and agents

ChIP-seq

Data deposition

☐ Confirm that both raw and final processed data have been deposited in a public database such as GEO.

☐ Confirm that you have deposited or provided access to graph files (e.g. BED files) for the called peaks.

Data access links<br>*May remain private before publication.*	*For "Initial submission" or "Revised version" documents, provide reviewer access links. For your "Final submission" document, provide a link to the deposited data.*
Files in database submission	*Provide a list of all files available in the database submission.*
Genome browser session<br>(e.g. UCSC)	*Provide a link to an anonymized genome browser session for "Initial submission" and "Revised version" documents only, to enable peer review. Write "no longer applicable" for "Final submission" documents.*

Methodology

Replicates	*Describe the experimental replicates, specifying number, type and replicate agreement.*
Sequencing depth	*Describe the sequencing depth for each experiment, providing the total number of reads, uniquely mapped reads, length of reads and whether they were paired- or single-end.*
Antibodies	*Describe the antibodies used for the ChIP-seq experiments; as applicable, provide supplier name, catalog number, clone name, and lot number.*
Peak calling parameters	*Specify the command line program and parameters used for read mapping and peak calling, including the ChIP, control and index files used.*
Data quality	*Describe the methods used to ensure data quality in full detail, including how many peaks are at FDR 5% and above 5-fold enrichment.*
Software	*Describe the software used to collect and analyze the ChIP-seq data. For custom code that has been deposited into a community repository, provide accession details.*

Flow Cytometry

Plots

Confirm that:

☒ The axis labels state the marker and fluorochrome used (e.g. CD4-FITC).

☒ The axis scales are clearly visible. Include numbers along axes only for bottom left plot of group (a 'group' is an analysis of identical markers).

☒ All plots are contour plots with outliers or pseudocolor plots.

☒ A numerical value for number of cells or percentage (with statistics) is provided.

Methodology

Sample preparation	FANS was only used for cell-sorting and not for immuno-fluorescence analysis (samples were used for sn-RNA-Seq).
Instrument	Beckman Coulter MoFloXDP cell sorter

Software	FlowJo v10.9
Cell population abundance	Nuclei were counted with a hemocytometer followed by flow cytometry (FCM) during sorting (source data for ED Fig.7).
Gating strategy	The sample was gated on the nuclei population based on the forward and side scatter densityFSC/SSC distribution, gated on SSC-W/SSC-H to get rid of the aggregates, gated on DAPI+ nuclei, further getting grid of aggregates, overlaid GFP+ with ungated population to verify the accuracy of nuclei gating.

☒ Tick this box to confirm that a figure exemplifying the gating strategy is provided in the Supplementary Information.

Magnetic resonance imaging

Experimental design

Design type	*Indicate task or resting state; event-related or block design.*
Design specifications	*Specify the number of blocks, trials or experimental units per session and/or subject, and specify the length of each trial or block (if trials are blocked) and interval between trials.*
Behavioral performance measures	*State number and/or type of variables recorded (e.g. correct button press, response time) and what statistics were used to establish that the subjects were performing the task as expected (e.g. mean, range, and/or standard deviation across subjects).*

Acquisition

Imaging type(s)	*Specify: functional, structural, diffusion, perfusion.*
Field strength	*Specify in Tesla*
Sequence & imaging parameters	*Specify the pulse sequence type (gradient echo, spin echo, etc.), imaging type (EPI, spiral, etc.), field of view, matrix size, slice thickness, orientation and TE/TR/flip angle.*
Area of acquisition	*State whether a whole brain scan was used OR define the area of acquisition, describing how the region was determined.*

Diffusion MRI ☐ Used ☐ Not used

Preprocessing

Preprocessing software	*Provide detail on software version and revision number and on specific parameters (model/functions, brain extraction, segmentation, smoothing kernel size, etc.).*
Normalization	*If data were normalized/standardized, describe the approach(es): specify linear or non-linear and define image types used for transformation OR indicate that data were not normalized and explain rationale for lack of normalization.*
Normalization template	*Describe the template used for normalization/transformation, specifying subject space or group standardized space (e.g. original Talairach, MNI305, ICBM152) OR indicate that the data were not normalized.*
Noise and artifact removal	*Describe your procedure(s) for artifact and structured noise removal, specifying motion parameters, tissue signals and physiological signals (heart rate, respiration).*
Volume censoring	*Define your software and/or method and criteria for volume censoring, and state the extent of such censoring.*

Statistical modeling & inference

Model type and settings	*Specify type (mass univariate, multivariate, RSA, predictive, etc.) and describe essential details of the model at the first and second levels (e.g. fixed, random or mixed effects; drift or auto-correlation).*
Effect(s) tested	*Define precise effect in terms of the task or stimulus conditions instead of psychological concepts and indicate whether ANOVA or factorial designs were used.*

Specify type of analysis: ☐ Whole brain ☐ ROI-based ☐ Both

Statistic type for inference (See Eklund et al. 2016)	*Specify voxel-wise or cluster-wise and report all relevant parameters for cluster-wise methods.*
Correction	*Describe the type of correction and how it is obtained for multiple comparisons (e.g. FWE, FDR, permutation or Monte Carlo).*

Models & analysis

n/a	Involved in the study
☐	☐ Functional and/or effective connectivity
☐	☐ Graph analysis
☐	☐ Multivariate modeling or predictive analysis

Functional and/or effective connectivity	*Report the measures of dependence used and the model details (e.g. Pearson correlation, partial correlation, mutual information).*
Graph analysis	*Report the dependent variable and connectivity measure, specifying weighted graph or binarized graph, subject- or group-level, and the global and/or node summaries used (e.g. clustering coefficient, efficiency, etc.).*
Multivariate modeling and predictive analysis	*Specify independent variables, features extraction and dimension reduction, model, training and evaluation metrics.*
